# Treatment of the nasal crease with a single phenol croton peel session

**DOI:** 10.1016/j.jdin.2024.08.020

**Published:** 2024-10-01

**Authors:** Gustavo Carneiro Nogueira, Raquel Iracema de Freitas Macedo Oliveira, Mariana Rocha Andrade, Bárbara Arze Rocha, Marina Vieira Rodrigues de Queiroz, Ana Carolina Tardin Rodrigues de Medeiros, Gisele Viana de Oliveira

**Affiliations:** a Dermatology Clinic, Itaúna, Minas Gerais, Brazil; b Medical Students, PROBIC - Science Initiative Student Program, Medical Sciences Faculty of Minas Gerais (FCMMG), Belo Horizonte, Minas Gerais, Brazil; c GV Dermatology, Belo Horizonte, Minas Gerais, Brazil; d Adjunct Post Graduation Professor, FCMMG, Belo Horizonte, Minas Gerais, Brazil; e Dermatology Department, Luxemburgo/Mario Penna Hospital, and Santa Casa de Misericórdia, Belo Horizonte, Minas Gerais, Brazil

**Keywords:** nasal crease, phenol croton peel

## Challenge

Nasal crease or nasal allergic crease is a very prevalent condition caused by the habit of rubbing the nose in upward motion with the hands or with fingers. Although its etiology is well defined, the literature lacks treatment options for this medical circumstance.

## Solution

**Fig 1:**
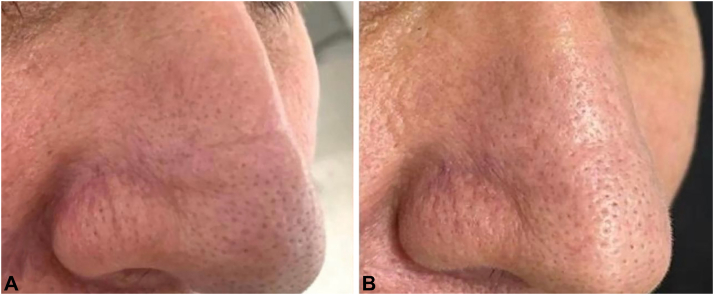
Nasal crease before and after treatment with only one session of the superlocalized phenol-croton peel: (**A**) Nasal crease before treatment in 45 degrees and (**B**) in front position. **A,** Nasal crease. **B,** Final outcome 1 year after the treatment.

We propose a treatment for the allergic crease^1^ using superlocalized phenol croton peel^2^ (Fig 1, Video 1, available on www.jaad.org). Cardiac safety is not a concern for this procedure, because it involves less than 0.5% of the body surface area (hand palm without the fingers).^3^ The dermatology team wears an N95 mask to avoid inhalation of the solution. The skin is degreased using acetone. The area of interest is marked using a white skin pencil, approximately 0.5 cm lateral to the crease in every direction. A 0.8% Hetter formula^2^ is applied thoroughly, over the entire area with the crease, and approximately 0.5 cm lateral to the crease, with a first pass using a wooden applicator with the wet tip wrapped in cotton. Thereafter, 5-10 multidirection passes are performed on the area of interest, without adding any extra solution. No analgesia is needed, because phenol itself works as an anesthetic drug.^2^ A large fan is used during the entire procedure against the patients face to avoid inhalation and to decrease the initial burning sensation, which only lasts for 10 seconds. The area is then covered with solid Vaseline. The patient is given a small portable fan to go home, because 10-20 minutes after the procedure a new burning sensation starts to last for about 2 hours in this region. The next day the patient is instructed to rinse the area, using a gentle cleanser solution and apply Vaseline after shower. The healing process generally takes 7 days in this region. We observe excellent outcomes and long term improvement over 2 years. A possible side effect is post inflammatory hyperpigmentation, which is transitory and rare in the nose area; however, the procedure should be restricted to phototypes up to Fitzpatrick 4. The “superlocalized-phenol-croton peel” is performed as an in-office procedure, does not require expensive technology and may lead to prolonged improvement.

## Conflicts of interest

None disclosed.

## Supplementary data

Video E1
